# Accessing the Past: A Sediment Core Revealing Anthropogenic Impacts of Technology-Critical Elements on the Marine Environment

**DOI:** 10.1007/s00244-024-01110-9

**Published:** 2025-01-17

**Authors:** Dominik Wippermann, Ole Klein, Hendrik Wolschke, Tristan Zimmermann, Anna Ebeling, Daniel Pröfrock

**Affiliations:** 1 Institute of Coastal Environmental Chemistry, Inorganic Environmental Chemistry, Helmholtz-Zentrum Hereon, Max-Planck Str. 1, 21502 Geesthacht, Germany; 2 Department of Chemistry, Inorganic and Applied Chemistry, Universität Hamburg, Martin-Luther-King-Platz 6, 20146 Hamburg, Germany; 3 Institute of Coastal Environmental Chemistry, Laboratory for Environmental Radiochemistry, Helmholtz-Zentrum Hereon, Max-Planck Str. 1, 21502 Geesthacht, Germany

## Abstract

One group of elements attracting more and more attention are so-called technology-critical elements (TCEs). In comparison with legacy pollutants, the anthropogenic impact of TCEs on the environment might still be minor, but various applications introduce them to the most remote places in the world including the marine environment. One area prone to pollution is the Baltic Sea, partly due to the lack of water exchange with the North Sea. In this study, a sediment core from the German Baltic Sea was used to analyze a total of 42 elemental mass fractions. Based on radiometric dating of ^210^Pb and ^137^Cs, results were classified in a recent (2020–2000) and a past period (< 1920), calculating background concentrations based on the Median + 2 Median Absolute Deviation (M2MAD) and the Tukey Inner Fence (TIF). Six legacy pollutants (Ni, Cu, Zn, As, Cd, Pb) and six TCEs (Ga, Ge, Nb, La, Gd, Ta) are discussed in detail. Anthropogenic impacts of both groups were assessed, and local enrichment factors were calculated showing an increase for the legacy pollutants (past period (≤ 0.8); recent period (≥ 1.2)), but also a minor increase for Ga, Ge and Nb (past period (0.9); recent period (1.1)). Values ≥ 1.5, indicating anthropogenic impact, were found for Cu, Zn, Cd and Pb, but also for Ge. Proposed background values may be considered as baseline for future studies.

**Graphical Abstract:**

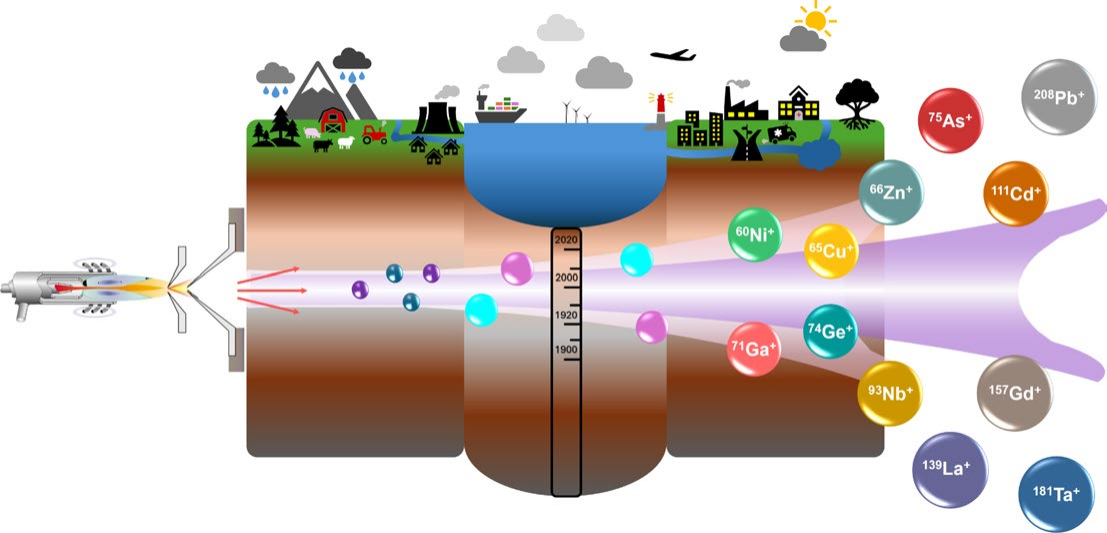

**Supplementary Information:**

The online version contains supplementary material available at 10.1007/s00244-024-01110-9.

Achieving and maintaining a good environmental status of aquatic systems is a critical goal for many legislators. Rigorous adherence to legal guidelines and threshold values for various pollutants is essential (*e.g.,* (Water Framework Directive 2000/60/EC 2000; Water Framework Directive 2008/56/EC 2008)). Within this context, legislative monitoring includes only selected, well-studied analytes. However, the release of new, potentially harmful substances needs to be considered. Among these, emerging environmental pollutants are technology-critical elements (TCEs) like Ga, Ge, Nb, In, Gd and Ta, which play a pivotal role in the generation of renewable energy, everyday products like computer and smartphones, communication technology and other highly specialized technological applications (Filella and Rodríguez-Murillo 2017; Nuss and Blengini 2018). Recent studies have demonstrated that galvanic anodes used as corrosion protection for offshore wind farms (OWF) release significant amounts of gallium (Ga) and indium (In) into the marine environment (Kirchgeorg et al. 2018; Reese et al. 2020; Ebeling et al. 2023).

In order to accurately assess and address the potential environmental impacts of these new emissions in terms of their ecotoxicology more information than the pure elemental mass fraction is required. Factors such as pH, grain size, and local biogeochemical processes also play a decisive role. Therefore, geochemical threshold values are often used to set legally binding limits for pollutants in order to identify elevated concentrations (Reimann et al*.* 2018). Thus, definition of background threshold or reference values for TCEs is required. Geological background thresholds are usually determined using methods such as the Median + 2 Median Absolute Deviation (M2MAD) or the Tukey Inner Fence (TIF). The M2MAD method tends to result in lower, more conservative thresholds, while the TIF method provides robust thresholds that are resistant to outliers within the dataset (Reimann et al*.* 2018).

As many TCEs are already released into the marine environment (*e.g.,* corrosion protection of OWFs, riverine inputs from urbanized areas), the accurate assessment of background values in surface sediments is often hindered. A solution to this problem is the analysis of sediment cores, which can be resolved, if long enough, into recent and anthropologically un-impacted parts.

Historically, the Baltic Sea has been a highly anthropogenically impacted area. It is estimated that up to 190 000 t of ammunition were dumped in the Skagerrak and the Baltic Sea after the end of World War II (WWII) (Bełdowski et al. 2020). Even today, the Baltic Sea is influenced by anthropogenic stress from shipping, fishing, (construction of) offshore structures and riverine inputs. Especially southwestern areas (Kattegat, Belt Sea, Kiel Bay and Mecklenburg Bay) are highly anthropogenically influenced (Korpinen et al. 2012). Indeed, aside various organic pollutants other legacy pollutants like Zn, Cd and Pb are regularly monitored by different agencies, but emerging contaminants like TCEs are often not considered (Bundesanstalt für Gewässerkunde 2024). Even though they are getting more attention recently, the applications causing the release of TCEs into the environment already exist (e.g., corrosion protection of OWFs), making it difficult to assess current anthropogenic inputs or to access background values based on surface sediment samples. Hereby, sediment cores allow the determination of mass fractions during the pre-industrial periods, allowing us to understand former conditions of marine sediment.

Therefore, this study presents data of a sediment core from the German Baltic Sea that was used to calculate reliable threshold values for a total of 42 elements, subdivided in a recent and an anthropogenically less impacted part, based on radiometric dating. A total of six legacy pollutants (Cu, Ni, Zn, As, Cd, Pb) and six TCEs (Ga, Ge, Nb, La, Gd, Ta) were selected and discussed in detail in the manuscript. Furthermore, local enrichment factors were calculated and presented to further assess potential accumulation of these elements within the investigated region. This study aims to present missing threshold values for different elements in the German Baltic Sea and to assess how different elemental mass fractions changed within marine sediment throughout the last century.

## Material and Methods

### Sampling

**Fig. 1:**
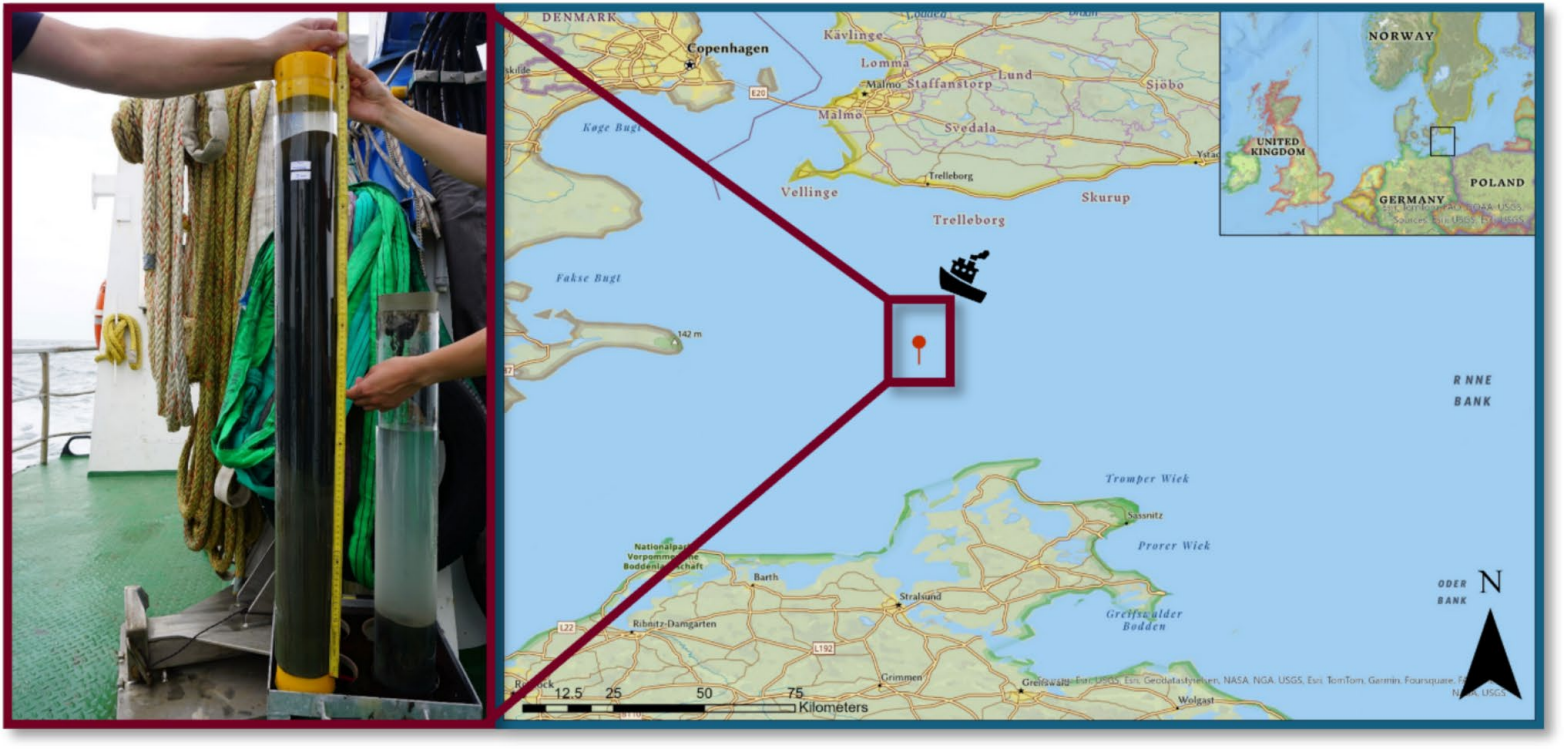
Sampling location of the sediment core taken in the German EEZ of the Baltic Sea in August 2022 (right) and picture of the core on board of the Ludwig Prandtl prior to slicing and freezing

A sediment core was taken in August 2022 as part of the sampling campaign LP202208 with the research vessel Ludwig Prandtl (Helmholtz-Zentrum Hereon) within the exclusive economic zone (EEZ) of the German Baltic Sea. The sediment core was taken by a Frahm Lot at 54.918° latitude and 13.136° longitude, with a total length of approximately 90 cm (see Fig. 1). Further sample preparation was conducted by slicing the core into 1 cm-thick slices and freezing on board, which resulted in 79 slices in total. Sampling equipment (plexiglass tube, plugs, stamp, and slicing equipment) was cleaned using type I reagent grade water (> 18.2 MΩ cm) in the laboratory prior to the sampling campaign and with demineralized water during work on the ship. Slices were collected in 120 mL polypropylene (PP) tubes, which were pre-cleaned in the laboratory by leaching with 1% (*w*/*w*) HNO_3_ for at least seven days followed by several rinsing steps using type I reagent grade water.

### Reagents and Standards

Laboratory work related to calibration and quantification was performed within a class 100 clean bench. Type I reagent grade water (> 18.2 MΩ cm) was obtained from an ultrapure water system consisting of an Elix 3 module (Merck Millipore, Darmstadt, Germany), a Milli-Q Element module (Merck Millipore, Darmstadt, Germany) and a Q-POD element (Merck Millipore, Darmstadt, Germany). HNO_3_ (65% *w*/*w*, Carl Roth, Karlsruhe, Germany) and HCl (37% *w*/*w*, Carl Roth, Karlsruhe, Germany) were further purified by double sub-boiling in perfluoralkoxy polymer (PFA)–stills (DST 4000 & DST 1000, Savillex, Minnesota, USA). Ultra-pure HBF_4_ (38% *w*/*w*, Chem-Lab, Zedelgem, Belgium) was used without further purification. Quantification was performed via external calibration, which was prepared from single element standards for Ti, Nb, In (Merck, Darmstadt, Germany) and custom-made multi-element calibration standards of varying composition for the remaining elements (Li, Al, Ca, Sc, V, Cr, Mn, Fe; Co, Ni, Cu, Zn, Ga, Ge, As, Rb, Sr, Y, Mo, Cd, Ba, La, Ce, Pr, Nd, Sm, Eu, Gd, Tb, Dy, Er, Ho, Tm, Yb, Lu, Ta, Tl, Pb, U) (Inorganic Ventures, Christiansburg, USA). All used standards are traceable to NIST.

For quality control purposes, an in-house standard “KUA-QC” based on multi-element solutions Multi VI (Merck, Darmstadt, Germany), ESI 33 and ESI 113 (Inorganic Ventures, Christiansburg, USA) and single element standards for Sc, Ti, Ge, Nb, In and Ta (Merck, Darmstadt, Germany) was used. Furthermore, the certified reference materials (CRM) GBW 07311 (marine sediment) (National Research Centre for Certified Reference Materials, Beijing, China) and BCR-2 (basalt rock) (Institute for Reference Materials and Measurements, Geel, Belgium) were used.

### Radiometric Measurements and Dating

Measurements were performed by the accredited Laboratory for Environmental Radiochemistry at the Helmholtz-Zentrum Hereon (D-PL-11208–01-00, DakkS) (Deutsche Akkreditierungsstelle 2021). Sample preparation for radiometric dating was carried out as described by Bunzel et al*.* (Bunzel et al. 2020). A freeze dried aliquot from every second slice, homogenized by milling, was sealed gas tide in a petri dish and stored for minimum 28 days for equilibrium of ^226^Ra with its daughter nuclides ^222^Rn, ^214^Pb and ^214^Bi. Measurement was performed by a high-purity low-level germanium detector (BE 3830P-7500SL-ULB Mirion Technologies (Canberra), Ruesselsheim, Germany). For calibration, an artificial CRM was prepared by doping a silica gel with reference solutions of ^137^Cs and ^226^Ra. (Eckert & Ziegler Nuclitec, Braunschweig, Germany). Measurement time varied between 90,000 and 600,000 s depending on the overall sample activity. Dating was based on ^210^Pb and ^137^Cs, as described in literature for dating marine sediments (Sanchez-Cabeza and Ruiz-Fernández 2012; Arias-Ortiz et al. 2018). Following the approach of Appleby and Oldfield ages and sedimentation rates were calculated from the supported ^210^Pb activity by means of the constant rate of supply (CRS) model and correlated by the fallout events of nuclear bomb test in 1963 and the Chernobyl accident 1986 (Appleby and Oldfield 1978). Details of application of the model can be obtained from literature (Sanchez-Cabeza and Ruiz-Fernández 2012; Arias-Ortiz et al. 2018; Bunzel et al. 2020). Supported ^210^Pb values were calculated based on the daughter nuclides of ^226^Ra (^214^Pb and ^214^Bi), the unsupported by measurement of ^210^Pb subtract by the supported value.

### Sample Digestion

Frozen samples were dried using a freeze dryer (Christ Gefriertocknungsanlagen, Osterode, Germany) and subsequently homogenized by a planetary ball mill (PM 400, Retsch, Haan, Germany). Selected slices were analyzed for their grain size distribution prior to milling using laser diffraction (Analysette 22 NanoTec, Fritsch, Idar-Oberstein, Germany). Samples were digested in triplicates of 50 mg each using 5 mL HNO_3_, 2 mL HCl and 1 mL HBF_4_, based on a microwave assisted acid digestion using a MARS 6 or MARS Xpress microwave (CEM Corp., Kamp Lintfort, Germany), following the protocol by Zimmermann et al*.* (Zimmermann et al. 2020). 55 mL TFM digestion vessels were pre-cleaned using an ETC EVO II (ANALAB, Hoenheim, France) acid vapor cleaner with HNO_3_ (65% *w/w*) and type I reagent grade water. CRMs GBW 07311 and BCR-2 were treated similarly and digested in triplicates per digestion batch. Blank samples were prepared in duplicates per digestion batch (12 samples). Digested samples were quantitatively transferred to 50 mL graduated, PP vessels (DigiTUBE®; SCP Science, Quebec, Canada) pre-cleaned with HNO_3_ (2% *w*/*w*) and filled to a total volume of 50 mL with type I reagent grade water.

### Instrumentation and Measurement Procedures

Sample digests were measured using an ICP-MS/MS (Agilent 8800, Agilent Technologies, Santa Barbara, California, USA) coupled to an ESI SC-4 DX FAST autosampler (Elemental Scientific, Omaha, Nebraska, USA) or to a prepFAST M5 system (Elemental Scientific, Omaha, Nebraska, USA). Detailed information about the operating parameters and cell gas modes of the ICP-MS/MS (Table A1), as well as quantified mass-to-charge ratios and their detection modes together with method validation parameters (corresponding element sheet) can be found in the Supplementary Information. H_2_, He and N_2_O were employed as cell gases, whereby H_2_ and N_2_O acted as reaction gases in MS/MS mode. Quantified mass-to-charge ratios and corresponding cell modes were selected based on achieved sensitivity, as well as by non-occurrence of isobaric and polyatomic interferences. Additional information about the working principles of ICP-MS/MS in combination with collision/reaction cell gases can be obtained from literature (Pröfrock and Prange 2012; Balcaen et al. 2015; Bolea-Fernandez et al. 2017; Klein et al. 2021; Lancaster et al. 2023). The instrument was tuned on a daily base to obtain optimal measuring conditions using a tune solution containing Li, Co, Y, Ce and Tl (10 µg L^−1^). External calibration covering a concentration range from 0 µg L^−1^ to 10,000 µg L^−1^ for Mg, Al, P, K, Ca, Ti, Mn, Fe, and Ba and 0 µg L^−1^ to 100 µg L^−1^ for the remaining elements, either automatically diluted by the prepFAST M5 system from two stock solutions, or manually prepared with eleven calibration standards covering the same concentration range but in conjunction with the ESI SC-4 DX FAST autosampler, as well as online dosed internal standards (10 µg L^−1^ Rh and Ir) were used for quantification. Potential carry-over effects were monitored by measuring wash blanks (2% HNO_3_ (*w*/*w*)) after each triplicate of samples. Calibration solutions were freshly prepared immediately before the measurement sequences.

### Data Processing and Calculations

MassHunter version 4.4 (Agilent Technologies, Tokyo, Japan) and a custom-written Excel^©^ (Microsoft, Redmond, Washington, USA) spreadsheet were used for processing multi-element data. Limits of detection (LOD) and limits of quantification (LOQ) were calculated based on procedural blanks, including three times the standard deviation (3 × SD) for LOD and ten times (10 × SD) for LOQ according to MacDougall et al*.* (MacDougall et al. 1980). Outliers were determined based on a Dean and Dixon outlier test (*P* = 0.95) and were excluded from further evaluation (Dean and Dixon 1951). Expanded uncertainties (*U*, *k* = 2) were calculated for each sample replicate according to Reese et al*.* based on a simplified Kragten approach, taking into account measurement precision of the instrument and repeatability of multiple sample digests (*n* = 3) (Kragten 1994; Reese et al. 2019). The uncertainty for each mass fraction and significant number of digits for each value are presented according to GUM and EURACHEM guidelines (Ellison and Williams 2012; Ellison et al. 2015). Figures were created using Origin® 9.8.0.200 (2021) (OriginLab Corporation, Northampton, Massachusetts, USA).

### Calculation of Local Enrichment Factors and Preliminary Reference Thresholds

The Baltic Sea sediment core was divided in a “recent” and a “past” section based on the results of the radiometric dating (see Results and Discussion). Based on this subdivision, reference thresholds were calculated following the approach of Reimann et al*.* as “M2MAD” and “TIF” as specified in the following.

$$\text{M}2\text{MAD}={10}^{\text{b}}$$

$$\text{where b}=\text{median}({\text{log}}_{10}\left({\text{X}}_{\text{i}}\right))+2*\text{MAD}({\text{log}}_{10}({\text{X}}_{\text{i}}))$$

$$\text{and MAD}=1.48\text{ median}({\text{x}}_{\text{i}}-\text{median}({\text{X}}_{\text{i}}))$$

$$\text{TIF}=\text{Q}3+1.5\text{ IQR}$$

*X*_*i*_ represents the mass fractions of the respective element and Q3 is the third quartile of the data set, while IQR is the interquartile range (Q3 – Q1) (Tukey 1977; Reimann et al. 2005; Reimann et al*.* 2018). Furthermore, local enrichment factors (LEF) were calculated for assessment of possible enrichment of elemental mass fractions throughout the sediment core. As described by Grygar et al*.* and Grygar and Popelka, the LEF was calculated as follows:

$${\text{LEF}}_{\text{X}}=\frac{\text{X}}{{\text{X}}_{\text{GBF}}}\,\text{ with }\,{\text{X}}_{\text{GBF}}=\text{f}(\text{Ti})$$

LEF sets the natural mass fraction (*X*_*GBF*_) of a target element *X* in relation to a suitable reference element (in this case Ti, *f*(Ti)) based on the average mass fraction of the reference element within each core slice (Grygar et al. 2014; Grygar and Popelka 2016). Ti was chosen as it showed the smallest coefficient of variation based on its mass fractions in the sediment core, and it does not (unlike Sc or Eu) reflect one of the element groups discussed in this study. Calculations can be found in Table A2 in the Supplementary Information.

## Results and Discussion

### Age and Grain Size of the Sediment Core

Analyzed core slices feature an unsupported ^210^Pb activity ranging between 293 ± 5 Bq kg^−1^ (1 cm) and 150 ± 4 Bq kg^−1^ (24 cm). Activity of the supported ^210^Pb (^226^Ra) varied between 25.1 ± 0.9 Bq kg^−1^ and 31.2 ± 0.6 Bq kg^−1^ within the same core section (1 cm–24 cm) followed by a decrease in unsupported ^210^Pb with 33 ± 3 Bq kg^−1^ at 26 cm (for further data see electronic supplementary Table A3). This indicates a disturbed zone with a loss of material, which covers approximately 25 cm to 44 cm. From 44 cm to the bottom of the core, the activity of unsupported ^210^Pb was below the limit of detection, indicating that the section of 45 cm to 79 cm is older than 100 years. The section of 1 cm to 23 cm can be dated to the time of 2022 to 2000. In the following evaluation, the sections are labeled “recent” for 1 cm to 23 cm and “past” for 45 cm to 79 cm, and the disturbed zone 24 cm–44 cm is excluded from further evaluation.

**Fig. 2:**
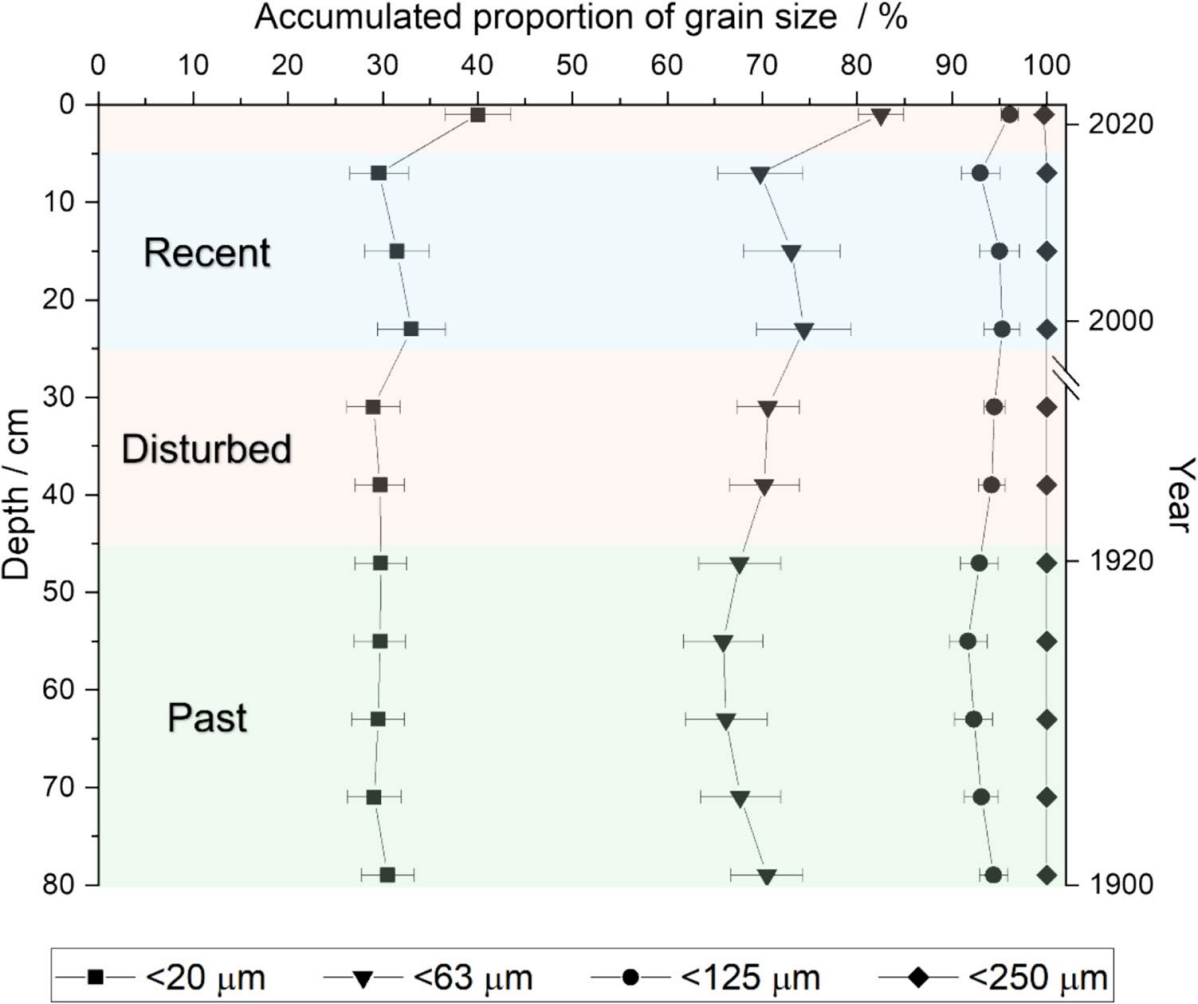
Subdivision of the sediment core based on the results of the radiometric dating and particle size analysis. The sections “recent” (blue, 5 cm–23 cm) and “past” (green, 45 cm – 79 cm) are used for further evaluation. Error bars correspond to the standard deviation of the mean, *n* ≥ 3

**Fig. 3:**
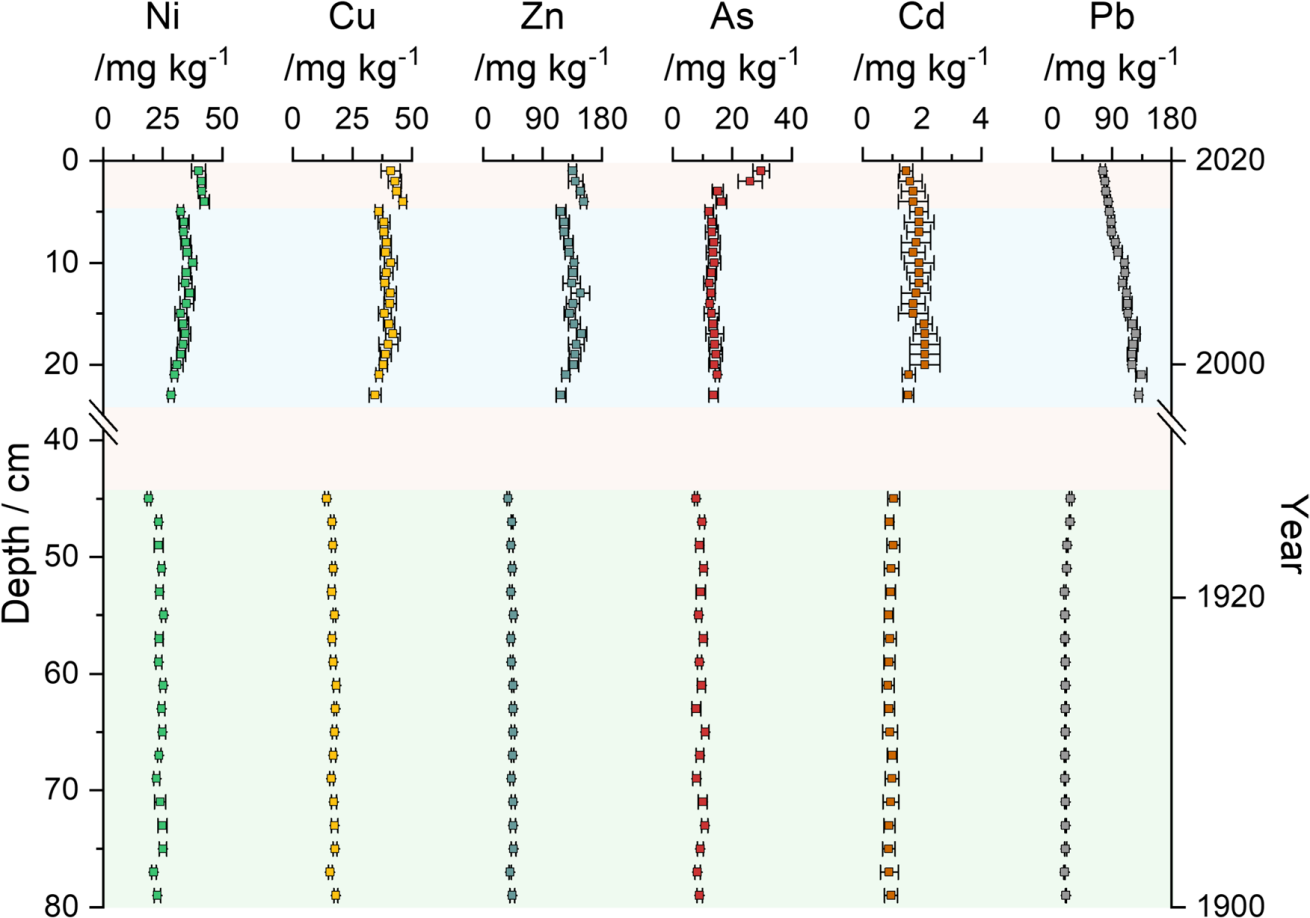
Metal mass fractions within the sediment core for selected legacy pollutants (Ni, Cu, Zn, As, Cd, Pb). Background color scheme: “recent” (blue, 5 cm–23 cm*) and “past” (green, *45 cm–79 cm), as well as “disturbed” (red). Error bars correspond to the expanded uncertainty *U(k* = *2*), *n* = *3*

Beside radiometric dating, the sediment core was characterized by particle size analysis of selected slices, to ensure that possible drifts in the mass fractions of an element are not influenced by changes in the fine grain fraction. Figure 2 shows the combined results of radiometric dating and particle size analysis and the resulting subdivision for further evaluation. Particle size distribution is within uncertainty of each other throughout the sediment core except for the < 20 μm and < 63 μm fractions within the first slice. As metals are preferably adsorbed to the smaller grain size fractions due to their relative high surface area, the top of the sediment core (1 cm–5 cm) was excluded from the “recent” section (Ackermann et al. 1983). Based on this characterization, the section “recent” (5 cm–23 cm) approximately reflects the time 2020 to 2000 and “past” (45 cm–79 cm) reflects the time 1920 and older (Figs. 2, 3).

### Legacy Pollutants

The selection of presented legacy pollutants (Ni, Cu, Zn, As, Cd, Pb) in this study was based on the availability of comparable data within literature, as well as various monitoring programs. Measured mass fractions in the sediment core are visualized in Fig. 3 and presented together with literature data in Table 1.

**Table 1:** Comparison of elemental mass fractions of selected legacy pollutants (Ni, Cu, Zn, As, Cd, Pb) from this study to data from literature.

	Further classification	Ni/mg kg^−1^	Cu/mg kg^−1^	Zn/mg kg^−1^	As/mg kg^−1^	Cd/mg kg^−1^	Pb/mg kg^−1^
This study EEZ-Germany	Recent (5 cm –23 cm)	27.3–39.2	37–45	111–157	10.7–15.6	1.36–2.6	83.6–142
	Past (45 cm –79 cm)	18.3–26.3	13.3–19.5	35.8–50	7.3–12.2	0.66–1.24	17.1–28.7
(Long et al. 1995)-	ERL	20.9	34	150	8.2	1.2	46.7
	ERM	51.6	270	410	70	9.6	218
(Borg and Jonsson 1996) Baltic Proper	Surface (0 cm–1 cm)	33–65	35–91	253–467	7–23	0.4–5.4	39–103
	pre-industrial (below 10 cm)	32–46	32–58	90–150	6–12	0.12–0.5	10–40
(Rudnick and Gao 2003) UCC	–	36–58	24–32	61–73	4.3–5.3	0.08–0.1	16.5–17.5
(Leipe et al. 2017) Southwestern Baltic Sea	German territorial waters	–	15.8–1200	39.4–1075	0.8–95.7	–	16.0–465
(Shahabi-Ghahfarokhi et al. 2021a) Baltic Proper	Core 12 (0 cm–40 cm) (2006–1938)	–	29–46	–	14 – 27	–	–
(Shahabi-Ghahfarokhi et al. 2021b) Baltic Proper		–	-	87.1–210	–	0.1–0.6	42.7–114
(Bundesanstalt für Gewässerkunde 2024) EEZ-Germany	OMBMPK7 (1995–2017)	31–113	35–297	144–1416	–	0.17–1.52	75–200

EEZ = exclusive economic zone; UCC = upper continental crust; ERL = effect range low; ERM = effect range median

#### Nickel

Mass fractions of Ni were in the range of 28.5 mg kg^−1^ ± 1.2 mg kg^−1^–37.6 mg kg^−1^ ± 1.6 mg kg^−1^ within the recent part of the sediment core and 19.1 mg kg^−1^ ± 0.8 mg kg^−1^–25.4 mg kg^−1^ ± 0.9 mg kg^−1^ within the past part. As shown in Fig.3, an increase in Ni mass fractions in the recent part of the sediment core is significant. Data from literature are within the same order of magnitude, but exceeds mass fractions presented in this study (see Table 1). Based on Long et al*.*, mass fractions within both core sections exceed the effect range low (ERL) of 20.9 mg kg^−1^, but are below the effect range median (ERM) of 51.6 mg kg^−1^, indicating the possibility of biological effects (Long et al. 1995).

#### Copper

Cu mass fractions showed a higher increase between the core sections than Ni with 34.5 mg kg^−1^ ± 2.5 mg kg^−1^–42 mg kg^−1^ ± 3 mg kg^−1^ in the recent part of the sediment core and 14.1 mg kg^−1^ ± 0.8 mg kg^−1^–18.2 mg kg^−1^ ± 1.3 mg kg^−1^ within the past part. Data from literature are within the same order of magnitude. Leipe et al*.* reported Cu mass fractions up to 1200 mg kg^−1^, but also investigated sampling areas closer to the coast (Leipe et al. 2017). Cu mass fractions within the past part fall below the ERL of 34 mg kg^−1^, whereas the recent part slightly exceeds them.

#### Zinc

Mass fractions of Zn between 118 mg kg^−1^ ± 7 mg kg^−1^ and 149 mg kg^−1^ ± 8 mg kg^−1^ were found in the recent part of the sediment core, which is more than twice compared to Zn mass fraction found in the past part (37.7 mg kg^−1^ ± 1.9 mg kg^−1^–46 mg kg^−1^ ± 4 mg kg^−1^). All values are below the ERL concentration of 150 mg kg^−1^ (Long et al. 1995). Mass fractions from literature are within the same order of magnitude (see Table 1). Exceptions (Zn mass fractions of > 1 g kg^−1^) were published by Leipe et al. and by an marine environmental database which probably represents sediments from near the coast. (Leipe et al. 2017; Bundesanstalt für Gewässerkunde 2024).

#### Arsenic

As mass fractions were above the ERL of 8.2 mg kg^−1^ within the recent part (12.2 mg kg^−1^ ± 1.5 mg kg^−1^–14.9 mg kg^−1^ ± 0.7 mg kg^−1^) and mainly above the ERL within the past part (7.8 mg kg^−1^ ± 0.5 mg kg^−1^–10.9 mg kg^−1^ ± 1.3 mg kg^−1^), indicating the possibility of biological effects (Long et al. 1995). Mass fractions from literature are within the same order of magnitude but tend to exceed mass fractions found in this study (see Table 1). Only Leipe et al*.* reported As mass fractions above the ERM (70 mg kg^−1^), apart from that mass fractions were closer to the ERL concentration in this study and within presented literature (Long et al. 1995; Leipe et al. 2017).

#### Cadmium

Cd mass fractions ranged between 0.86 mg kg^−1^ ± 0.2 mg kg^−1^ and 1.05 mg kg^−1^ ± 0.19 mg kg^−1^ in the past part and from 1.54 mg kg^−1^ ± 0.18 mg kg^−1^ to 2.1 mg kg^−1^ ± 0.5 mg kg^−1^ in the recent part (see Table 1). Borg and Jonsson published Cd mass fractions up to 5.4 mg kg^−1^ within the upper part of their sediment core, which exceeds the recent part of this study (Borg and Jonsson 1996). Regular monitoring studies report Cd mass fractions of 0.17 mg kg^−1^–1.52 mg kg^−1^, which is in good agreement with data from the past part of the sediment core. Therefore, especially the recent part of the sediment core tends to show elevated Cd mass fractions and in contrast to the past part, also exceeds the ERL of 1.2 mg kg^−1^, but is still significantly lower than the ERM of 9.6 mg kg^−1^ (Long et al. 1995).

#### Lead

Pb featured the highest increase in mass fractions between the past section (18 mg kg^−1^ ± 0.9 mg kg^−1^–27 mg kg^−1^ ± 1.7 mg kg^−1^) and the recent section (86.1 mg kg^−1^ ± 2.5 mg kg^−1^–134 mg kg^−1^ ± 8 mg kg^−1^). The “pre-industrial” Pb mass fractions published by Borg and Jonsson (10 mg kg^−1^–40 mg kg^−1^) are in good agreement with the one from the past section of this study (Borg and Jonsson 1996). The upper part of the core from Borg and Jonsson, as well as data published by Shahabi-Ghahfarokhi et al*.*, are in good agreement with the Pb mass fractions within the recent part of the sediment core (see Table 1). Leipe et al*.* (up to 465 mg kg^−1^) and the monitoring program of the Bundesanstalt für Gewässerkunde (up to 200 mg kg^−1^) reported mass fractions exceeding the ones presented in this study (Leipe et al. 2017; Bundesanstalt für Gewässerkunde 2024). Overall the ERL of 46.7 mg kg^−1^ is exceeded in almost all data sets, but only data published by Leipe et al*.* exceeds the ERM of 218 mg kg^−1^ (Long et al. 1995).

#### Threshold Values for Legacy Pollutants

Based on the presented data, preliminary threshold values were calculated based on the M2MAD and TIF approach following Reimann et al*.* for the selected legacy pollutants (see Table 2) (Reimann et al*.* 2018). For further data and threshold values not discussed in the manuscript, the reader is referred to the Supplementary Information (corresponding element sheet). Calculated threshold values increased between the past and recent part of the sediment core. We assume that this is mainly caused by anthropogenic inputs during 1920 – 2000, which separates the two sections. This allows us to present current, realistic threshold values and background values that are significantly less affected by anthropogenic inputs.

**Table 2:** Threshold values for selected legacy pollutants (Ni, Cu, Zn, As, Cd, Pb) calculated from the recent and past part of the sediment core following the M2MAD and TIF approach based on Reimann et al*.*(Reimann et al*.* 2018)

	Recent		Past	
	M2MAD	TIF	M2MAD	TIF
Ni / mg kg^−1^	37.5	38.4	26.5	26.9
Cu / mg kg^−1^	42.1	43.4	18.6	18.9
Zn / mg kg^−1^	152	156	47.0	49.0
As / mg kg^−1^	15.1	15.5	11.6	11.8
Cd / mg kg^−1^	2.5	2.5	1.0	1.1
Pb / mg kg^−1^	141	151	22	22

Ni and Cu show comparable mass fractions in both parts of the sediment core. Both elements play a vital role in high-tech applications of modern-day life. Cu is mainly used within the electronic industry and is essential for the electricity supply (International Copper Association 2024). The focusing on renewable energy will further increase the demand for Cu in the upcoming years (Buchholz and Brandenburg 2018). Ni is mainly used in stainless steel alloys, but also plays an essential role in battery cell production, which is also influenced by the expansion of renewable energies (Nickel Institute 2024). Overall, both elements play a key role within the development of industrialized countries (Henckens and Worrell 2020). Therefore, we assume that anthropogenic inputs of both elements into the marine environment are to be expected.

Threshold values for Zn show a threefold increase between past and recent. Nevertheless, threshold values for the recent part are in good agreement with most literature values (see Table 1), which even exceed values of this study in some cases. Zn is introduced into the marine environment through riverine inputs from urbanized areas but also from corrosion protection of offshore windfarms, ships and/or harbors (Kirchgeorg et al. 2018; Reese et al. 2020). In contrast, the upper part of the recent section shows a decrease in Zn mass fractions, indicating decreasing Zn inputs. (see Fig. 3).

Arsenic is a known threat to the marine environment of the Baltic Sea in part due to the massive dumping of ammunition and chemical warfare agents after WWII (Bełdowski et al. 2020). The release of organo-arsenic compounds from these containers depends on their status of corrosion, which is a complex procedure based on many local factors (HELCOM 2013; Vanninen et al. 2020). As mass fractions from this study are above the ERL, indicating the possibility of biological effects. It has already been shown that warfare-related As compounds have accumulated in marine biota samples (Niemikoski et al. 2017). Indeed, there are other known anthropogenic sources for As (e.g., mining, fossil fuel burning, wood preservatives) (Kalia and Khambholja 2015). As the sampling site is not known for dumped ammunition and mass fraction ranges overlap within both core sections, we do not assume warfare-related As accumulation within the sediment.

Cd mass fractions in this study exceed the data based on regularly monitoring by authorities (Bundesanstalt für Gewässerkunde 2024). Cd is used in different applications causing anthropogenic inputs, like mining and metal production, fertilizer production, paints and pigments, but also in corrosion protection of ships (Kubier et al. 2019; Xu et al. 2021). We assume that the two most plausible anthropogenic Cd inputs affecting the Baltic Sea are the release by corrosion protection of ships and riverine inputs. Furthermore, the Baltic Sea is known for anoxic areas, which have increased throughout the last century (Carstensen et al. 2014; Kuliński et al. 2022). Within these anoxic conditions, Cd forms bisulfide complexes with a high affinity to bind to fine particles and clay-rich sediments (Öztürk 1995; Pohl and Hennings 1999; Shahabi-Ghahfarokhi et al. 2021b). This would explain the higher mass fractions of this study compared to regular monitoring, as these anoxic zones can act like local hotspots. Overall, if anoxic zones in the Baltic Sea further increase, higher mass fractions of Cd might be found in future studies.

Pb showed the strongest increase in mass fraction and subsequently within the calculated threshold values. Nevertheless, the thresholds values are in good agreement to literature values (see Table 1). A major anthropogenic source of Pb in the 20th century was leaded fuel, which led to the accumulation of Pb in the environment. Due to the disturbed zone of the core, the maximum Pb mass fraction was likely not observed. Shahabi-Ghahfarokhi et al*.* and Logemann et al*.* reported decreasing Pb mass fractions within the 1980s after worldwide restriction during the 1970s and 1980s. Furthermore, Logemann et al*.* were able to relate the increase in Pb mass fractions to leaded fuel additives based on Pb isotope measurements (Shahabi-Ghahfarokhi et al. 2021b; Logemann et al. 2022). Another major source for anthropogenic Pb are coal combustion processes (Komárek et al. 2008). With guidelines and laws further regulating anthropogenic Pb inputs, we assume a further decrease in Pb mass fractions.

### Technology-Critical Elements

**Fig. 4:**
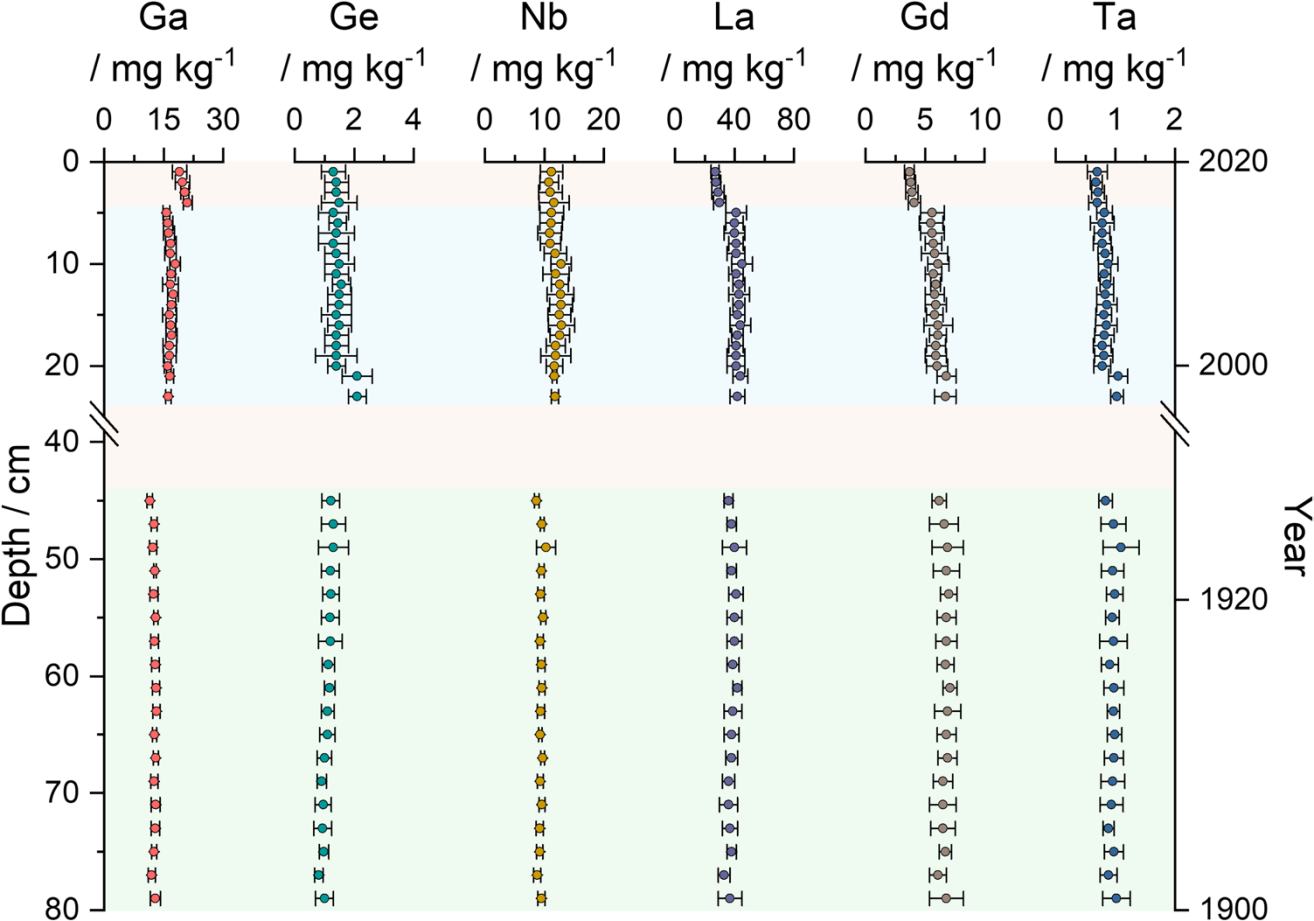
Metal mass fraction within the sediment core for selected TCEs (Ga, Ge, Nb, La, Gd, Ta). Background color scheme: “recent” (blue, 5 cm – 23 cm) and “past” (green, 45 cm – 79 cm), as well as “disturbed” (red). Error bars correspond to the expanded uncertainty *U*(*k* = 2), *n* = 3

The selection of presented TCEs (Ga, Ge, Nb, La, Gd, Ta) is based on the classification of Filella et al*.* for less-studied TCEs (LSTCE), which covers Ga, Ge, Nb and Ta. Environmental background concentrations for these elements is particularly scarce (Filella and Rodríguez-Murillo 2017). La and Gd were chosen as representatives of the rare earth elements (REE), La as a light REE (LREE) and Gd as a heavy REE (HREE). Measured mass fractions in the sediment core are visualized in Fig. 4 and presented together with comparable data from literature in Table 3. Based on the limited data availability of TCEs (especially LSTCEs) and the lack of consideration in official monitoring programs, comparable data were summarized in a broader range also considering different geographic areas and different sediment types (lake, river). The results of each element are presented and discussed below, followed by a general classification of all six elements together with the presentation of the calculated threshold values (Fig. 4).

**Table 3:** Comparison of elemental mass fractions of selected technology-critical elements (Ga, Ge, Nb, La, Gd, Ta) from this study to data from literature.

	Further classification	Ga/mg kg^−1^	Ge/mg kg^−1^	Nb/mg kg^−1^	La/mg kg^−1^	Gd/mg kg^−1^	Ta/mg kg^−1^
This study EEZ-Germany	Recent (5 cm–23 cm)	14.8–19.2	0.8–2.6	8.9–15.1	33–52	4.5–7.4	0.58–1.21
	Past (45 c–79 cm)	10.8–14.1	0.67–1.8	8.3–11.9	29–45	5.4–7.8	0.73–1.4
(Rudnick and Gao 2003) UCC	**–**	16.8–18.2	1.3–1.5	11–13	28–34	3.7–4.3	0.8–1.0
(Ohta et al. 2010) Japan	Coastal sea sediment	2.2–20.9	**–**	0.70–28.4	2.89–30.9	0.82–5.53	0.01–2.07
(Ingri et al. 2014) Baltic Sea	Bothnian Bay Upper part (0–1000 years), Core 1	–	Approx.1.3–1.7				
(Bačić et al. 2021) Croatia	Lake sediment	0.19–19.8	0.01.–1.5	0.19–17.4	0.44–42.3	–	–
(Klein et al. 2022a) German North Sea	Marine sediment,offshore (North)	14–23	1.4–2.3	11.0–15.4	18.7–41.9	4.6–7.8	0.8–1.2
(Klein et al. 2022b) German Rhine	River sediment	8.0–15.14	1.6–8.5	8.9–25.5	21.5–156	4.0–9.5	0.77–1.8

EEZ = exclusive economic zone; UCC = upper continental crust

#### Gallium

Mass fractions of Ga were in the range of 15.7 mg kg^−1^ ± 0.9 mg kg^−1^–17.9 mg kg^−1^ ± 1.3 mg kg^−1^ within the recent part of the sediment core and 11.5 mg kg^−1^ ± 0.7 mg kg^−1^–13.2 mg kg^−1^ ± 0.9 mg kg^−1^ within the past part. Both mass fraction ranges are in good agreement with comparable literature values (see Table 3) and in good agreement with upper crust values of 16.8 mg kg^−1^–18.2 mg kg^−1^ (Rudnick and Gao 2003). Selected publications reported mass fractions lower than this study. Ohta et al*.* and Bačić et al., reported Ga mass fractions as low as 2.2 mg kg^−1^ and 0.19 mg kg^−1^, respectively. Lowest measured Ga mass fraction of this study was 11.5 mg kg^−1^ ± 0.7 mg kg^−1^ (Ohta et al. 2010; Bačić et al. 2021).

#### Germanium

Ge mass fractions were about an order of magnitude lower than Ga, ranging between 1.3 mg kg^−1^ ± 0.5 mg kg^−1^ and 2.1 mg kg^−1^ ± 0.5 mg kg^−1^ within the recent part of the sediment core and 0.81 mg kg^−1^ ± 0.14 mg kg^−1^ and 1.3 mg kg^−1^ ± 0.5 mg kg^−1^ within the past part, indicating a slight increase in Ge mass fractions. Indeed, both mass fraction ranges in good agreement with upper crust levels of 1.3 mg kg^−1^–1.5 mg kg^−1^ (Rudnick and Gao 2003). Comparable data are available for the Baltic Sea. Ingri et al*.* reported Ge mass fractions of approximately 1.3 mg kg^−1^–1.7 mg kg^−1^, which is in accordance to this study (Ingri et al. 2014). Klein et al*.* published Ge mass fractions up to 8.5 mg kg^−1^ in river sediments from the German Rhine, which feature a higher anthropogenic load compared to a offshore sampling side and mass fractions of 1.4 mg kg^−1^–2.3 mg kg^−1^ for marine sediments from the German North Sea, which is in good agreement to this study. (Klein et al. 2022b, 2022a).

#### Niobium

Nb showed a slight increase in mass fractions with 10.9 mg kg^−1^ ± 2.0 mg kg^−1^–12.9 mg kg^−1^ ± 2.2 mg kg^−1^ in the recent part of the sediment core and 8.7 mg kg^−1^ ± 0.4 mg kg^−1^–10.3 mg kg^−1^ ± 1.6 mg kg^−1^ in the past part. This is in good agreement to other literature values (see Table 3) and the geogenic background of 11 mg kg^−1^–13 mg kg^−1^ (Rudnick and Gao 2003). Ohta et al*.* and Klein et al*.* published Nb mass fractions up to 28.4 mg kg^−1^ and 25.5 mg kg^−1^, respectively (Ohta et al. 2010; Klein et al. 2022b).

#### Lanthanum

Within uncertainty, La mass fractions showed no increase with 40 mg kg^−1^ ± 7 mg kg^−1^–45 mg kg^−1^ ± 7 mg kg^−1^ in the recent part of the sediment core and 33 mg kg^−1^ ± 4 mg kg^−1^–42 mg kg^−1^ ± 3 mg kg^−1^ in the past one. Overall, mass fractions are slightly above the geogenic background of 28 mg kg^−1^–34 mg kg^−1^, but in good agreement with comparable literature values (see Table 3) (Rudnick and Gao 2003). Klein et al*.* published La mass fractions up to 156 mg kg^−1^, which is attributed to anthropogenic inputs from a catalyst production facility near Worms located in the Upper Rhine region (Rhine river km 447.3), as also suggested by Kulaksız and Bau (Kulaksız and Bau 2011, 2013; Klein et al. 2022b). For marine sediments from the German North Sea, Klein et al*.* reported La mass fractions ranging from 18.7 mg kg^−1^–41.9 mg kg^−1^, which are slightly lower than data from this study but within the same order of magnitude.

#### Gadolinium

Gd showed a slight decrease in mass fractions with 5.5 mg kg^−1^ ± 1.0 mg kg^−1^–6.8 mg kg^−1^ ± 0.8 mg kg^−1^ in the recent part of the sediment core and 6.1 mg kg^−1^ ± 0.7 mg kg^−1^–7.1 mg kg^−1^ ± 0.6 mg kg^−1^ in the past part. Indeed, both values overlap within uncertainty. Mass fractions of this study are slightly above the geogenic background of 3.7 mg kg^−1^–4.3 mg kg^−1^, but otherwise consistent with literature values (see Table 3) (Rudnick and Gao 2003). Klein et al*.* reported slightly higher Gd mass fractions in the German North Sea and German Rhine (up to 7.8 mg kg^−1^ and up to 9.5 mg kg^−1^, respectively), which is possibly impacted by anthropogenic Gd inputs, originating from the use of gadolinium-based contrast agents, which are discharged via waste water treatment facilities into rivers (Rhine) and through major tributaries subsequently into the marine environment (North Sea) (Brünjes and Hofmann 2020; Klein et al. 2022a, 2022b).

#### Tantalum

Mass fractions of Ta were in the range of 0.78 mg kg^−1^ ± 0.20 mg kg^−1^–1.05 mg kg^−1^ ± 0.16 mg kg^−1^ within the recent part of the sediment core and 0.84 mg kg^−1^ ± 0.11 mg kg^−1^–1.1 mg kg^−^1 ± 0.3 mg kg^−1^ within the past part. Ta mass fractions were stable throughout the sediment core. Literature reports similar mass fractions for Ta, which also matches the geogenic background of 0.8 mg kg^−1^–1.0 mg kg^−1^ (Rudnick and Gao 2003).

#### Threshold values for Technology-Critical Elements

Following Reimann et al*.* (see Table 4), threshold values were calculated for TCEs based on the M2MAD and TIF approach (Reimann et al*.* 2018). For further data and threshold values not discussed in the manuscript, the reader is referred to the Supplementary Information (corresponding element sheet). Ga, Ge and Nb showed minor increases in the recent part, whereas Ge and Nb overlap with the past part within uncertainties. La, Gd and Ta showed stable mass fractions within both core sections. Based on these finding, we expanded the calculated threshold values by combining both parts of the sediment core (5 cm–23 cm and 45 cm – 79 cm), allowing the coverage of a larger data set.

**Table 4:** Threshold values for selected technology-critical elements (Ga, Ge, Nb, La, Gd, Ta) calculated from the recent and past part of the sediment core following the M2MAD and TIF approach from Reimann et al*.*(Reimann et al*.* 2018)

	Recent		Past		Combined	
	M2MAD	TIF	M2MAD	TIF	M2MAD	TIF
Ga / mg kg^−1^	17.6	17.7	13.4	13.5	21.4	22.4
Ge / mg kg^−1^	1.6	1.7	1.5	1.5	1.8	1.8
Nb / mg kg^−1^	14.1	14.0	9.9	10.1	15.0	16.0
La / mg kg^−1^	43.0	46.0	43.0	44.0	45.0	48.0
Gd / mg kg^−1^	6.5	6.6	7.1	7.4	8.0	8.0
Ta / mg kg^−1^	0.94	0.96	1.03	1.04	1.15	1.20

Ga became important throughout the last decades especially for high-tech applications within the electronic industry and for renewable energy (e.g., by Ga containing photovoltaic or solar panels) (Butcher and Brown 2014). Ga primary production has evolved rapidly from approximately 100 t in 2000 to more than 300 t in 2020 (US Geological Survey 2001, 2021c). Within the marine environment anthropogenic, Ga inputs caused by the dissolution of galvanic anodes used for corrosion protection of offshore windfarms have been reported (Kirchgeorg et al. 2018; Reese et al. 2020). This specific input might be able to increase the anthropogenic load of Ga in the Baltic Sea. Indeed, mass fractions found throughout the recent part of the sediment core do not indicate an enrichment during the period 2000—2020. Therefore, we assume that the increase in Ga mass fraction is based on a longer time scale and might be connected to the production of Al throughout the last century. Ga, as part of the main Al-ore *bauxite*, is concentrated in the industrial waste from aluminum refineries (red mud) (Løvik et al. 2015). Von der Au et al*.* reported increased Ga mass fractions in sediment samples of the Elbe River of 33 μg kg^−1^ in 2014 compared to 14 μg kg^−1^ in the 1980s (Au et al. 2022). Mass fractions of this study might be explained by evolved environmental standards in Europe and less Al producing facilities due to high energy prices.

Ge and Nb feature slightly increased mass fractions within the recent part of the sediment core but overlap within their uncertainties compared to the past part. Both elements featured comparable production rates of 130 t of Ge and 78 t Nb in 2020 (Nb production decreased in 2020, compared to 2019: 97 t) (US Geological Survey 2021a, 2021b). Ge is mainly used in highly specialized technology like fiber-optic systems, infrared optics, as catalyst and within electric applications like solar panels, which adds up to a total of 95% of the used Ge in 2010 (Melcher and Buchholz 2014). Due to these specialized applications and the fact that the main production of Ge is not located in countries bordering the Baltic Sea, we assume that there is no significant anthropogenic input of Ge within the analyzed sediment core. Primary application of Nb is the production of high strength, low alloy steel, which is used in many different areas, including the construction of ships (Linnen et al. 2014). This might act as an anthropogenic source of Nb to the marine environment like the Baltic Sea. Even though ships use corrosion protection systems like Zn-based galvanic anodes, ships are still exposed to corrosion, which would explain the minor increase in Nb mass fractions compared to Zn. Furthermore, corrosion of shipwrecks would also contribute to anthropogenic Nb emissions. Based on the application of Nb, we would assume that (future) anthropogenic inputs are more likely for Nb than for Ge.

La, Gd and Ta showed no increase in mass fractions, indicating no current anthropogenic inputs. Known anthropogenic sources of La include catalyst production facilities. Indeed, these are limited point sources, due to the particle active behavior of LREE, which causes La to accumulate in the upper sediment layer (Kulaksız and Bau 2013). Gd is commonly used as a stable chelate complex in contrast agents in magnetic resonance imaging (MRI). Based on the high stability of these complexes and the inability of most water treatment plants to remove them, Gd is especially introduced into the aquatic environment in urbanized areas (Brünjes and Hofmann 2020). Gd tends to stay dissolved in the water body as complexed by its original ligand or by formation of new complexes (*e.g.,* carbonates) (Verplanck et al. 2005). As a result, current anthropogenic inputs of Gd can only be found to a very small extent in the sediment.

The total mass of Ta used in industry is low compared to other elements (world production of Ta is around 2 t a^−1^) (US Geological Survey 2021a). Its main use is in the electronic industry (*e.g.,* capacitor) and other high specialized applications in the field of medicine or aircraft, but its overall demand rises only to a very small extend due to increased performance in industry and miniaturization of many electronic products (Linnen et al. 2014). Based on its low natural background, anthropogenic Ta inputs could be identified early, but so far, we do not see measurable inputs in the sediment of the Baltic Sea.

### Assessment of Potential Elemental Mass Fraction Enrichment

To further assess the mass fraction changes throughout the sediment core, LEFs were calculated based on the approach of Grygar et al*.* and Grygar and Popelka using Ti as a reference element (Grygar et al. 2014; Grygar and Popelka 2016). Ti was chosen as it showed the smallest coefficient of variation based on its mass fractions in the sediment core, and it does not (unlike Sc or Eu) reflect one of the element groups discussed in this study. An overview of the calculated LEFs can be found in Table 5; for further information, the reader is referred to Table A2 in the Supplementary Information.

**Table 5:** Local enrichment factors (range and median) for legacy pollutants (Ni, Cu, Zn, As, Cd, Pb) and technology-critical elements (Ga, Ge, Nb, La, Gd, Ta) calculated from the recent and past part of the sediment core following Grygar et al*.* and Grygar and Popelka (Grygar et al. 2014; Grygar and Popelka 2016)

	Recent		Past	
	Range	Median	Range	Median
*Legacy pollutants*				
Ni	1.0–1.3	1.2	0.7–0.9	0.8
Cu	1.2–1.5	1.4	0.5–0.7	0.6
Zn	1.3–1.6	1.5	0.4–0.5	0.5
As	1.0–1.3	1.2	0.7–1.0	0.8
Cd	1.0–1.5	1.3	0.6–0.8	0.7
Pb	1.3–1.9	1.7	0.3–0.4	0.3
*Technology-critical elements*				
Ga	1.1–1.2	1.1	0.8–0.9	0.9
Ge	1.0–1.5	1.1	0.6–1.0	0.9
Nb	1.0–1.2	1.1	0.8–0.9	0.9
La	1.0–1.1	1.0	0.8–1.1	1.0
Gd	0.9–1.0	0.9	1.0–1.1	1.1
Ta	0.8–1.1	0.9	1.0–1.2	1.1

Especially the legacy pollutants feature a significant increase in the median LEFs between the past part (≤ 0.8) and the recent part (≥ 1.2), compared to the TCEs. Based on the classification of Birch and Olmos, LEFs of 1.5–3, 3–5, 5–10 and > 10 are considered as evidence of minor, moderate, severe and very severe anthropogenic impact, respectively (Birch and Olmos 2008). Considering this classification, the legacy pollutants (except Ni and As) feature LEFs above 1.5, while Zn and Pb even show median LEFs > 1.5 within the recent part, indicating minor anthropogenic impact. This underlines the importance of ongoing monitoring and regulatory limit values. For TCEs, all median LEFs are in the range of 0.9—1.1 (recent and past part), indicating no anthropogenic pressure so far. Nevertheless, for Ga, Ge and Nb the LEFs increased from 0.9 to 1.1 between past and recent part indicating minor accumulation. Furthermore, Ge features LEFs of up to 1.5, which would represent a minor impact. Since this was observed for two core slices, followed by 16 slices in the range of 1.0–1.2, we assume this to be a limited increase and not a general anthropogenic input. Overall, we can observe slightly increasing trends also affecting TCEs.

## Summary and Conclusion

In this work, a sediment core taken in August 2022 within the German Baltic Sea was used to evaluate and assess the anthropogenic impact of 42 elements. Based on radiometric dating using ^210^Pb and ^137^Cs, the sediment core was subdivided in two sections reflecting the time frames of 2020–2000 and before 1920. Multi-element analysis showed a significant increase in mass fractions in the recent part of the sediment core, especially for legacy pollutants like Ni, Cu, Zn, As, Cd and Pb. TCEs like Ga, Ge, Nb, La, Gd and Ta featured only minor to no increase in mass fractions. Calculation of LEFs confirmed these findings showing minor anthropogenic impact (LEF ≥ 1.5) for Cu, Zn, Cd and Pb. LEFs of TCEs were on average below 1.5 indicating no anthropogenic impact so far. Yet mass fractions of nine of the discussed elements (Ni, Cu, Zn, Ga, Ge, As, Nb, Cd, Pb) increased compared to the past part of the sediment core. Overall, this study presents robust and recent threshold values for a total of 42 elements, allowing other studies to compare and assess their findings. Furthermore, 12 selected elements were discussed for anthropogenic impacts and their potential origins.

Based on the results, future work should consider a broader range of elements for regular monitoring programs, especially as robust and precise multi-element methods have become available. Threshold values presented in this study may act as a baseline for future investigations of pathways and fates of different inorganic contaminants within the German Baltic Sea, which is a powerful tool as advancing technological applications introduce emerging contaminants into new areas. Furthermore, we emphasized the importance of studies like this, due to the lack of comparable data for many emerging contaminants, which are essential for a better understanding of anthropogenic impacts on land–river–sea systems in the near future.

## Supplementary Information

Below is the link to the electronic supplementary material.

Supplementary file1 (XLSX 253 kb)
